# Clip-and-snare method with a pre-looping technique versus conventional method in the treatment of precancerous lesion and early gastric cancer: a retrospective study

**DOI:** 10.1186/s12876-024-03231-w

**Published:** 2024-05-17

**Authors:** Ruichong Deng, Jiatong Wu, Defeng Li, Benhua Wu, Ruiyue Shi, Yanhui Tian, Jun Yao, Li-sheng Wang

**Affiliations:** Department of Gastroenterology, The Second Clinical Medical College, The First Affiliated Hospital, Shenzhen People’s Hospital, Jinan University, Southern University of Science and Technology, No.1017, Dongmen North Road, Luohu District, Shenzhen, 518020 China

**Keywords:** Precancerous lesion, Early gastric cancer, Endoscopic submucosal dissection, Clip-and-snare method with a pre-looping technique

## Abstract

**Background:**

Low grade intraepithelial neoplasia (LGIN) and high grade intraepithelial neoplasia (HGIN) are potential precancerous lesion of gastric neoplasms. Endoscopic submucosal dissection (ESD) is the first option for the treatment of precancerous lesion and early gastric cancer (EGC). Traction is an effective method to improve efficiency, and reduce complications during ESD. In this study, we shared a useful traction method using the clip-and-snare method with a pre-looping technique (CSM-PLT) for precancerous lesion and EGC.

**Methods:**

We retrospectively analyzed patients received ESD combined with CSM-PLT or conventional ESD from June 2018 to December 2021 in Shenzhen People’s hospital. The primary outcome was resection speed.

**Results:**

Forty-two patients were enrolled in ESD combined with CSM-PLT group and sixty-five patients in conventional ESD group respectively. Baseline characteristics were comparable among two groups (*P*>0.05). There were no significant differences in terms of R0 resection rate, *en* bloc resection rate (97.6% vs. 98.5%, *P* = 1.000 and 97.6% vs. 96.9%, *P* = 1.000, respectively), operation costs (933.7 (644.1-1102.4) dollars vs. 814.7 (614.6-988.3) dollars, *P* = 0.107), and hospital stays (8.0 ± 3.1 days vs. 7.3 ± 3.2 days, *P* = 0.236). In addition, no significant difference was observed with respect to complications (*P*>0.05). However, the resection speed of ESD combined with CSM-PLT was faster than that of conventional ESD (11.3 (9.4–14.9) mm^2^/min vs. 8.0 (5.8–10.9) mm^2^/min, *P* < 0.001), particularly lesions located in anterior wall and lesser curvature. In addition, the association between ESD combined with CSM-PLT and resection speed was still supported after propensity matching scores (PMS).

**Conclusions:**

CSM-PLT can help to improve ESD efficiency without reducing the *en* bloc resection rate or increasing the incidence of complications.

## Introduction

Low grade intraepithelial neoplasia (LGIN) and high grade intraepithelial neoplasia (HGIN) could develop into gastric cancer. Endoscopic submucosal dissection (ESD) is the standard therapy for precancerous lesions and early gastric cancer (EGC) [1]. It is possible to achieve high *en* bloc resection rate through various modifications regardless of tumor size or location, as well as improve the accuracy of pathological diagnosis and reduce recurrent rate [2, 3]. However, ESD is still a challenging technique, because it is time-consuming and difficult to manipulate. At the same time, it’s not easy to obtain a clear visual field, which may result in complications such as perforation and bleeding [4, 5]. Besides, studies showed that the lesions at gastric fundus and body are risk factors of ESD perforation, because the gastric wall in such area are thinner than that in the lower portion [6–8]. Therefore, effective devices and techniques are needed to reduce complications and shorten the operation time during the procedure.

Traction methods are novel devices and techniques that facilitate ESD procedure [9]. It helps to create tension in the vertical direction, which fully exposes the submucosal layers and vascular distribution, thus ensuring a good endoscopic visual field, accelerating the operation process and achieving accurate cutting [10, 11]. So far, different kinds of traction methods have been reported in ESD, such as clip and line [2, 12], S-O clip [3, 13], magnetic beads [14] and elastic band [15]. However, these methods have some disadvantages more or less, such as uncontrollable traction tension or direction, high cost, and the need of additional devices, so these traction methods have not been routinely applied in the clinical practice [9]. Hence, a traction method with low cost, simple device and easy operation is in need of assisting ESD.

The clip-and-snare method is a novel traction method emerged in recent years. It was first reported by Baldaque-Silva et al. [16] and was named as “yo-yo technique”. Yoshida et al. [17] further improved this method by prelooping the snare around the transparent cap from outside the endoscope, which effectively solved the uncontrollable-position and difficult operation, and named it as clip-and-snare method with a pre-looping technique (CSM-PLT). CSM-PLT has been performed in esophageal, gastric and colorectal ESD. It can not only control the tension but also the direction of traction (push or pull), thereby fully exposing the submucosal layer and lesion edge, improving cutting efficacy and procedural safety [17–19]. However, few studies have compared the effectiveness and safety between ESD combined with CSM-PLT and conventional ESD. Therefore, the present study is to assess the safety and feasibility comparing ESD combined with CSM-PLT with conventional ESD for the treatment of LGIN or HGIN or EGC at gastric fundus and body.

## Methods

### Patients

**Fig. 1:**
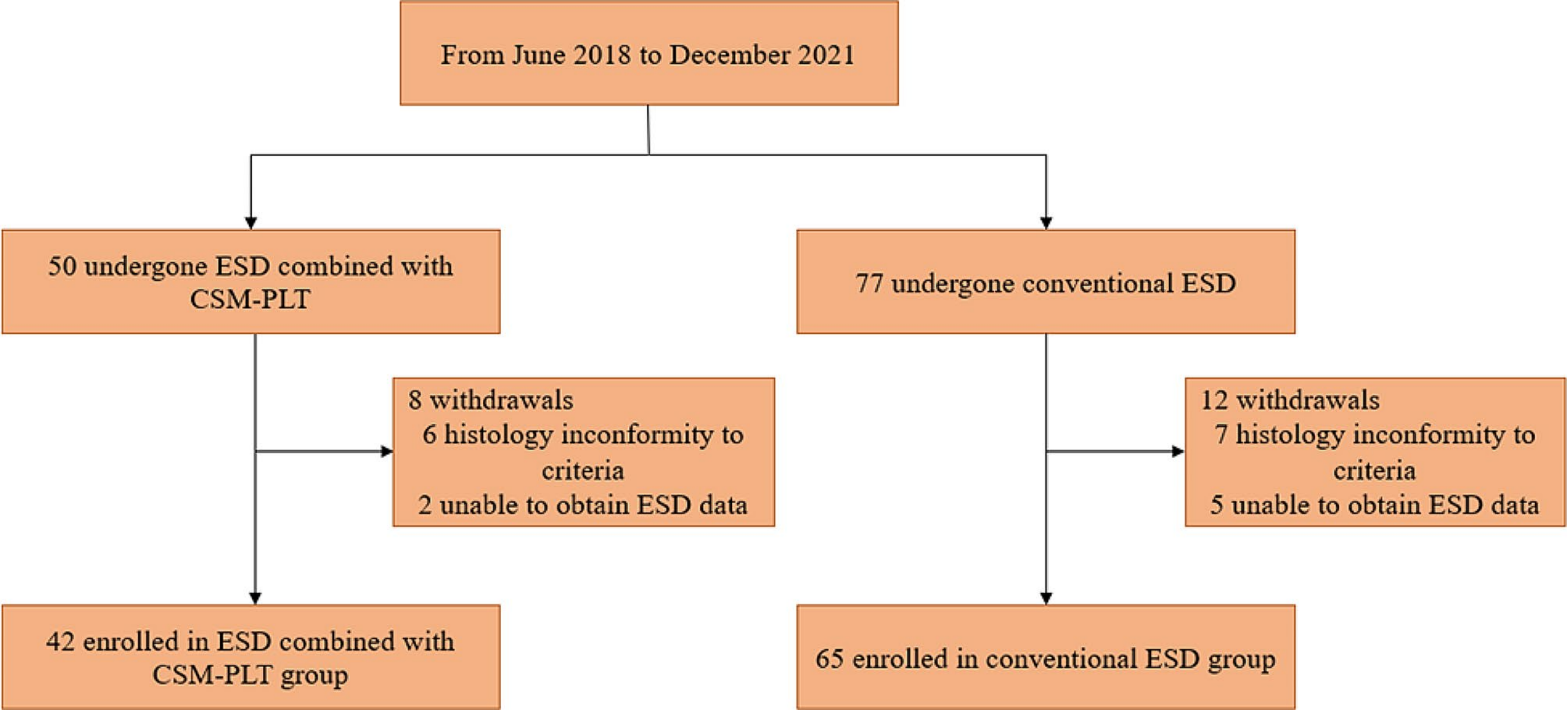
Flow chart of the inclusion

This was a retrospective, single-center experience conducted in Shenzhen People’s hospital (The Second Clinical Medical College, Jinan University). A total of 50 patients with precancerous lesions or EGC at gastric fundus or body undergone ESD combined with CSM-PLT in our hospital from September 2019 to December 2021. The inclusion criteria were: (1) Aged 18–80 years; (2) Preoperative diagnosis of ESD was LGIN or HGIN; (3) Pathological and endoscopic diagnosis was differentiated intramucosal gastric cancer with no ulcer before ESD; (4) The lesion was located at gastric fundus or body; (5) The maximum diameter of the lesion was more than 2 cm. The exclusion criteria included: (1) severe cardiopulmonary disease; (2) coagulation dysfunction (international normalized ratio >2.0, platelet count <100,000/mm^3^); (3) unable to obtain the data of ESD procedure, for instance, lesion size, operation time and specimen area. Based on the exclusion criteria, a total of 42 patients were eligible for this study. Correspondingly, a total of 77 patients with LGIN or HGIN or EGC at gastric fundus or body undergone conventional ESD from June 2018 to September 2019, among whom 65 patients met the inclusion criteria were enrolled (Fig. 1). All ESD were performed by two endoscopists (Ben-hua Wu and Li-sheng Wang) with more than 5 years of experience.

### ESD combined with CSM-PLT (with video)

**Fig. 2:**
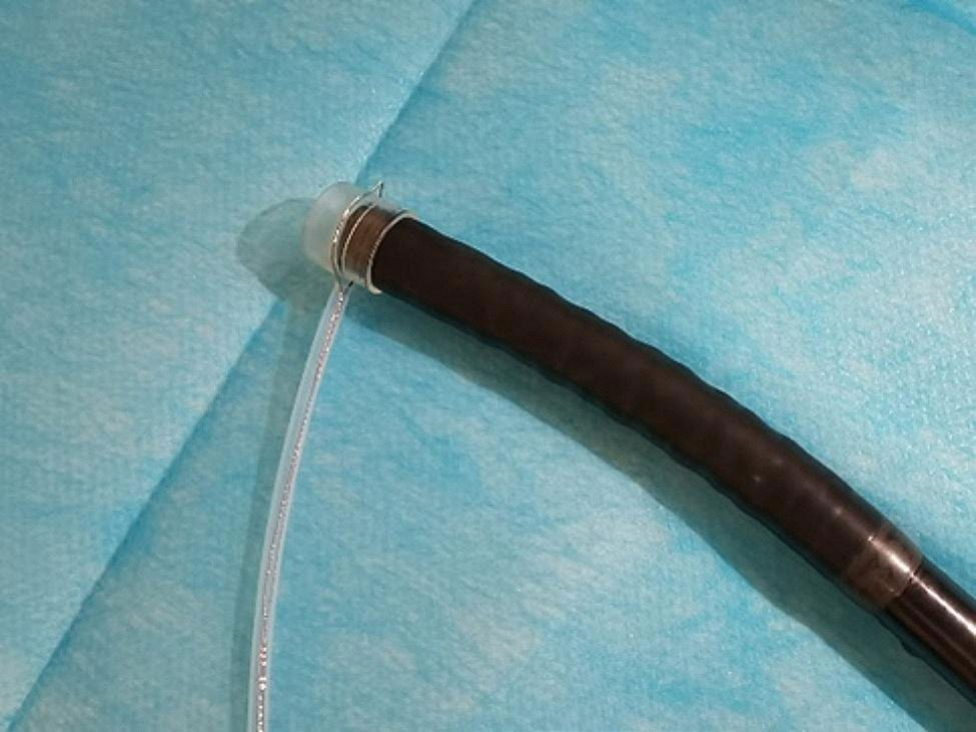
A single-channel endoscope with a disposable transparent cap, and a snare was looped around it

**Fig. 3:**
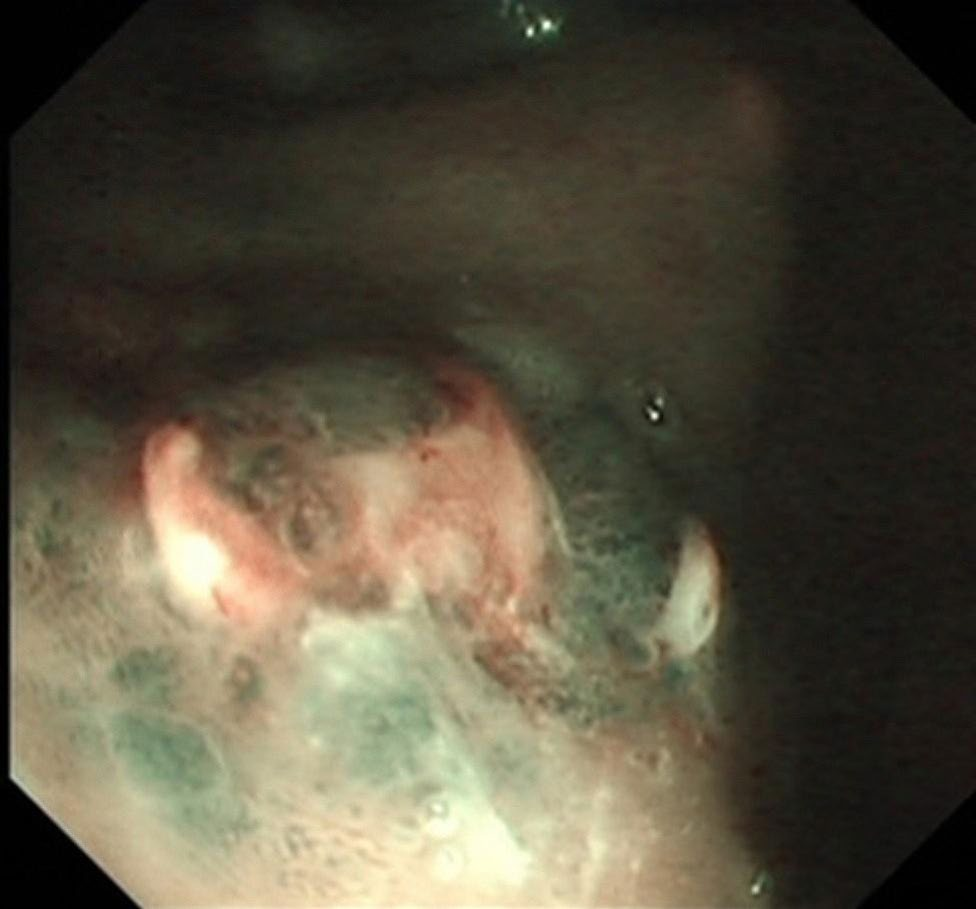
Lesion located in posterior wall of gastric fundus under narrow band imaging (NBI)

**Fig. 4:**
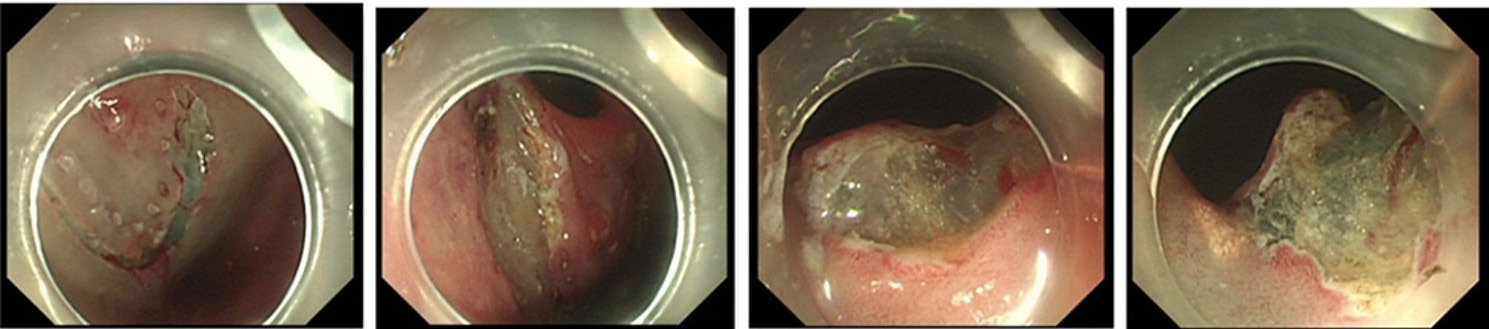
Make a circumferential incision outside the dots, and dissect the lesion from oral side to anal side to make a mucosal flap

**Fig. 5:**
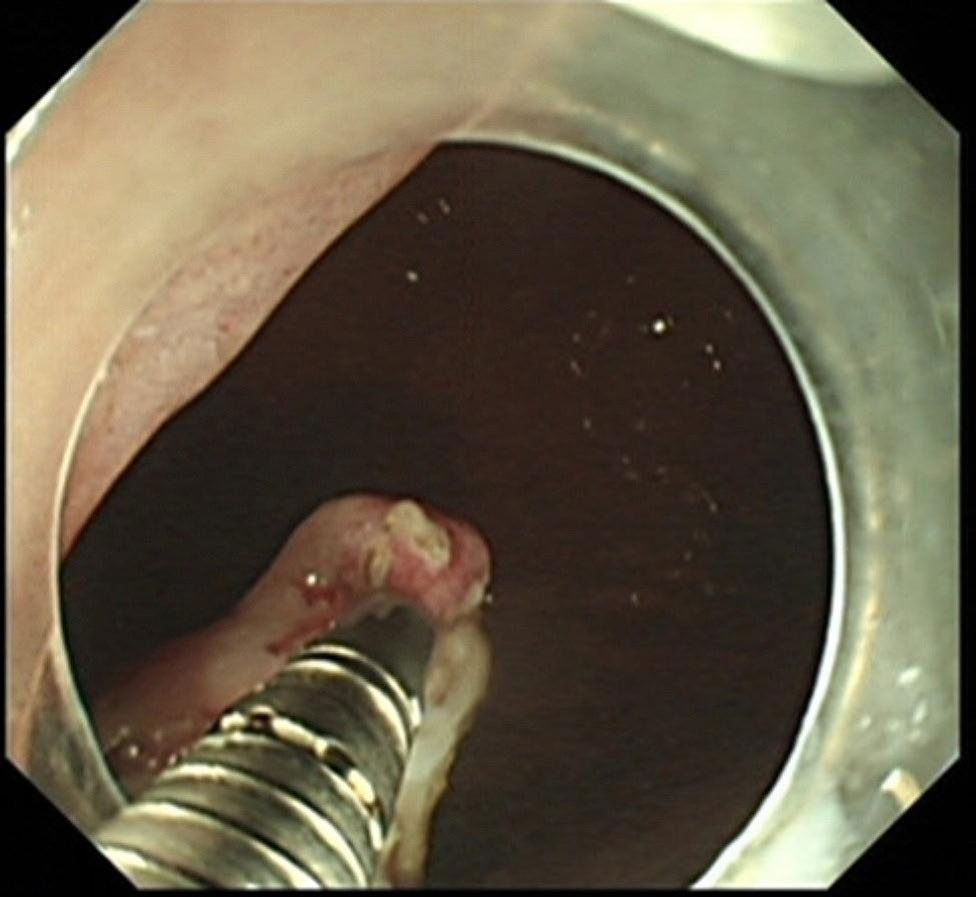
A hemoclip was used to grasp the mucosal flap from the oral side of the lesion

**Fig. 6:**
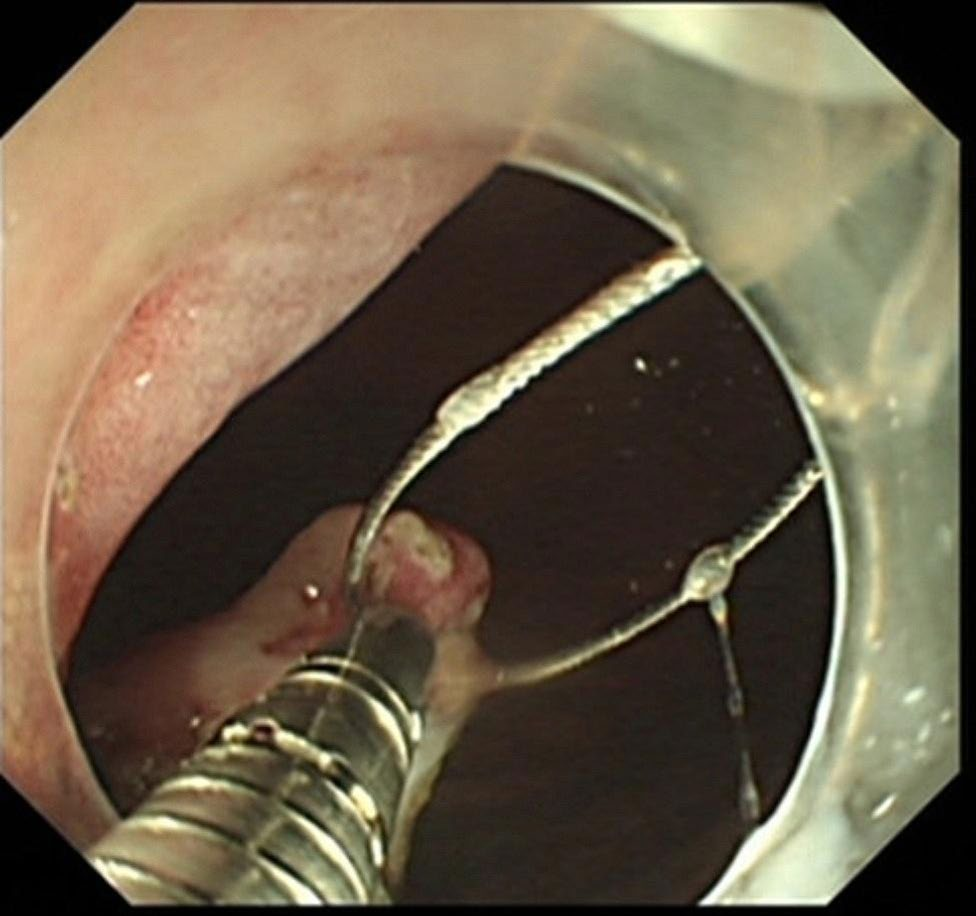
The pre-looped snare was loosened from the transparent cap and moved it toward hemoclip, and then tightened the base of hemoclip

**Fig. 7:**
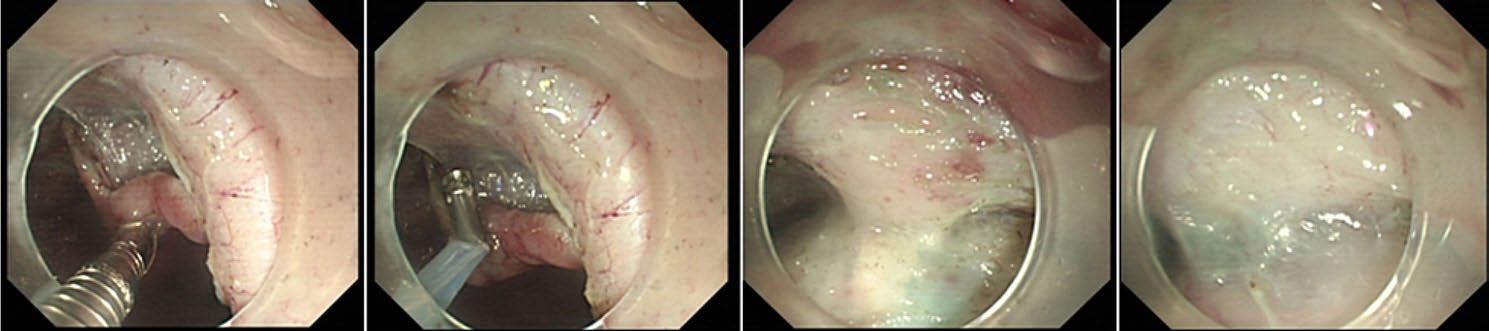
Push or pull the snare to create traction that fully exposed the layers of submucosa

**Fig. 8:**
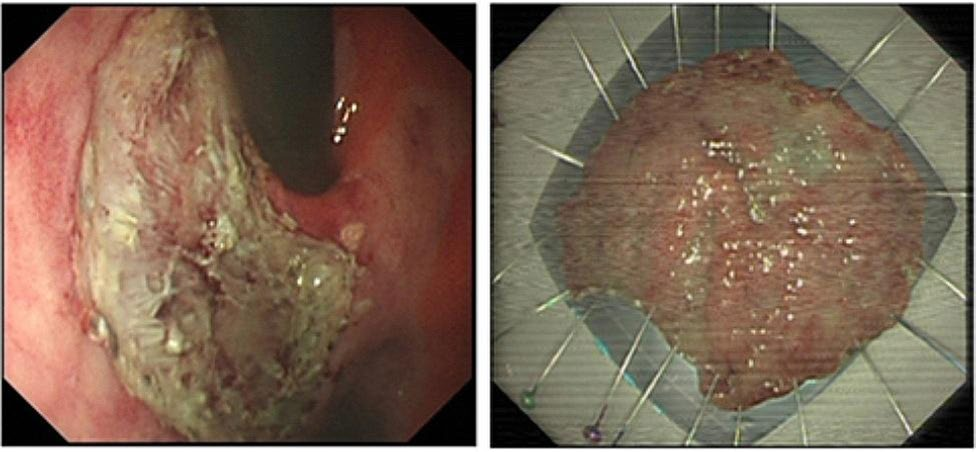
The operative wound and specimen (The pathological diagnosis was differentiated intramucosal carcinoma)

ESD combined with CSM-PLT was performed under tracheal intubation and general anesthesia. We adopted a single-channel endoscope (GIF-Q260J; Olympus Co, Tokyo, Japan) equipped with a disposable transparent cap (D-201-11804; diameter 12.4 mm, length 4 mm; Olympus Co, Tokyo, Japan) on the endoscopic tip (Fig. 2). The main steps of ESD combined with CSM-PLT were performed as follows: (1) Mark dots on about 0.5 cm outside the edge of lesions using Dual knife (KD-650 L; Olympus Co, Tokyo, Japan); Electronic surgical workstation: VIO 200D, ERBE, Germany; Operating mode: FORCED COAG model, effect 2; Maximum power 25 W. (Fig. 3). (2) Repeated submucosal injection was performed outside the dots using saline containing 0.3% indigo carmine, until the lesion was significantly elevated. (3) Make a circumferential incision outside the dots with Dual knife. After circumferential incision, dissect the lesion from oral side to anal side with Dual knife to make the mucosal flap (Endocut I mode, FORCED COAG model, effect 2, maximum power 50 W, VIO 200D, ERBE, Germany). (Fig. 4) (4) The endoscope was withdrawn, and a snare (MICRO.TECH, Nanjing, China) was looped around the transparent cap on the outside of the endoscope (Fig. 2). (5) Reinsert the endoscope and snare, and a rotatable repeated opening and closing hemoclip (MICRO.TECH, Nanjing, China) was inserted through the endoscope channel, which was used to grasp the mucosal flap from the oral side of the lesion (Fig. 5). (6) The pre-looped snare was loosened from the transparent cap and moved the snare toward hemoclip, and then tightened the base of hemoclip (Fig. 6). (7) The hemoclip was released from the hemoclip deployment device, and then the delivery part of hemoclip was withdrawn through the endoscope channel. (8) Push or pull the snare to expose vascular distribution and the submucosa (Fig. 7), use Dual knife to dissect the lesion, and then fix the specimen with a pin for pathological examination (Fig. 8). (9) Intra-operative bleeding during ESD or the visible blood vessels on the surface of the wound was performed soft coagulation (SOFT COAG mode, effect 4, maximum power 80 W, VIO 200D, ERBE, Germany) with a hot biopsy forceps (FD-410LR; Olympus Co, Tokyo, Japan).

### Outcomes and definitions

The primary outcome was resection speed. Secondary outcome included *en* bloc resection rate, R0 resection rate, hospital stay, operation costs and complications. Specimen area was calculated by ellipse formula: specimen area = 3.14 × (longer axis length /2) × (shorter axis length /2). Procedural duration was measured as the time from the start of submucosal injection to the end of cutting submucosal fiber. Resection speed was defined as the ratio of specimen area to procedural duration. *En* bloc resection was defined as the removal of the lesion in one piece. R0 resection was defined as pathological negative at the resection edge of the lesion (horizontal and vertical). Hospital stay was defined as cumulative days from admission to discharge. Operation cost was defined as the costs of gastric ESD. Complications included intra-operative bleeding, post-operative bleeding, intra-operative perforation and post-operative perforation. Intra-operative bleeding was defined as hemorrhage during ESD that requires endoscopic intervention. Post-operative bleeding was defined as hematemesis, melena, or a decrease in hemoglobin of more than 2 g/dl after ESD. Intra-operative perforation was defined as the visualization of abdominal structure during ESD procedure. Post-operative perforation was defined as perforation occurring after the day of ESD.

### Statistical analysis

Categorical variables were presented as frequency and percentage (%), and were compared using Chi-square test or Fisher’s exact test. Continuous variables were presented as mean ± standard deviation (SD) or median and interquartile range (IQR) based on the distribution, and were compared using student’s t-test or Mann-Whitney U test. All analyses were performed by the statistical software PASW 18.0 (SPSS Inc, Chicago, IL, USA). All P values were two-sided, and a P value <0.05 was considered statistically significant.

## Result

### Baseline characteristics

From June 2018 to December 2021, a total of 42 patients in ESD combine with CSM-PLT were eligible, and 65 patients undergone conventional ESD in the same period were enrolled in this study. There were no significance differences in terms of gender, age, lesion location or position (*P* = 0.096, *P* = 0.760, *P* = 0.694, *P* = 0.921, respectively) between two groups. The pathological outcome showed that most of the lesions were low grade intraepithelial neoplasia (LGIN) (66.7% vs. 78.5%, *P* = 0.294) (Table 1).

**Table 1:** Baseline characteristics

Characteristics	ESD combined with CSM-PLT (*n* = 42)	Conventional ESD (*n* = 65)	P value
Sex (%)			0.096^a^
Male	34(81.0)	43(66.2)	
Female	8(19.0)	22(33.8)	
Age (years)	57.4 ± 9.5	58.1 ± 11.4	0.760^b^
Location (%)			0.694^a^
Fundus	16(38.1)	23(35.4)	
Body	26(61.9)	42(64.6)	
Position (%)			0.921^a^
Anterior wall	9(21.4)	14(21.5)	
Posterior wall	15(35.7)	21(32.3)	
Greater curvature	4(9.5)	9(13.8)	
Lesser curvature	14(33.3)	21(32.3)	
Histology (%)			0.294^a^
Adenocarcinoma	7(16.7)	5(7.7)	
LGIN	28(66.7)	51(78.5)	
HGIN	7(16.7)	9(13.8)	

^a^Pearson chi-square test

^b^Student’s t-test

LGIN, low grade intraepithelial neoplasia; HGIN, high grade intraepithelial neoplasia

### Clinical outcomes

The R0 resection rate was comparable between two groups (97.6% vs. 98.5%, *P* = 1.000). No significant difference was observed with respect to *en* bloc resection rate between two groups (97.6% vs. 96.9%, *P* = 1.000). There was no significance difference with regard to hospital stays (8.0 ± 3.1d vs. 7.7 ± 3.5d, *P* = 0.647) and operation costs (933.7 (644.1-1102.4) dollars vs. 814.7 (614.6-988.3) dollars, *P* = 0.107) in these two groups. The specimen area of the ESD combine with CSM-PLT group was larger than that of conventional ESD group (1206.9 (561.3-1972.3) mm^2^ vs. 510.2 (336.4-861.5) mm^2^, *P* = 0.000), and the procedural duration of conventional ESD group was shorter than that of ESD combine with CSM-PLT group (60.0 (47.5-100.5) min vs. 86.5 (60.0-148.5) min, *P* = 0.017). Moreover, the resection speed of ESD combined with CSM-PLT was significantly faster than that of conventional ESD (11.3 (9.4–14.9) mm^2^/min vs. 8.0 (5.8–10.9) mm^2^/min, *P* = 0.000). In addition, the association between ESD combined with CSM-PLT and resection speed was still supported after propensity matching scores (PMS) (Table 2).

**Table 2:** Clinical outcomes

Variable	Crude model			PSM model		
	ESD combined with CSM-PLT (*n* = 42)	Conventional ESD (*n* = 65)	P value	ESD combined with CSM-PLT (*n* = 32)	Conventional ESD (*n* = 32)	P value
Specimen area (mm^2^)	1206.9(561.3-1972.3)	510.2(336.4-861.5)	**0.000** ^**c**^	734.9(461.5-862.4)	698.8(438.7-865.6)	0.052^c^
Procedural duration (min)	86.5(60.0-148.5)	60.0(47.5-100.5)	**0.017** ^**c**^	56.7(43.2–90.6)	59.7(45.8–99.7)	**0.049** ^**c**^
Resection speed (mm^2^/min)	11.3(9.4–14.9)	8.0(5.8–10.9)	**0.000** ^**c**^	10.2(8.6–13.2)	7.3(4.7–9.2)	**0.032** ^**c**^
R0 resection (%)	41(97.6)	64(98.5)	1.000^a^	30(100)	55(100)	1.000^a^
En bloc resection (%)	41(97.6)	63(96.9)	1.000^a^	30(100)	55(100)	1.000^a^
Hospital stays (days)	8.0 ± 3.1	7.3 ± 3.2	0.236^b^	7.5 ± 2.8	7.4 ± 2.7	0.836^b^
Operation costs (dollars)	933.7(644.1-1102.4)	814.7(614.6-988.3)	0.107^c^	903.6(634.6-1009.8)	806.8(605.7-954.9)	0.367^c^
Complications (%)						
Intra-operative bleeding	20(47.6)	37(56.9)	0.346^a^	18(56.3)	17(53.2)	1^a^
Post-operative bleeding	1(2.4)	1(1.5)	1.000^d^	0	0	N/A
Intra-operative perforation	1(2.4)	2(3.1)	1.000^e^	0	0	N/A
Post-operative perforation	0	0	N/A	0	0	N/A

*Note*^a^ Pearson chi-square test, ^b^ Student’s t-test, ^c^ Mann-Whitney test, ^d^ Fisher’s exact test, ^e^ Continuity correction. The significance of bold emphasis is *P*<0.05

#### Complications

One patient experienced intra-operative perforation in ESD combined with CSM-PLT group, whereas there were two patients developed into intra-operative perforation in conventional ESD group (2.4% vs. 3.1%, *P* = 1.000). No post-operative perforation was observed in two groups. Intra-operative bleeding occurred in 20 patients in ESD combined with CSM-PLT group and 37 patients in conventional ESD group (47.6% vs. 56.9%, *P* = 0.346). Only one post-operative bleeding was observed in ESD combined with CSM-PLT groups, and there was one post-operative bleeding occurred in conventional ESD group (2.4% vs. 1.5%, *P* = 1.000). Fortunately, the perforation was clipped by hemoclips under endoscopy in time, while the delay-bleeding were performed endoscopic intervention, and the patients recovered well after the operation. All intra-operative bleeding was successfully intervened with hot biopsy forceps.

### Subgroup analysis

We performed a subgroup analysis comparing resection speed by lesion location, position and histology. As shown in Table 3, the resection speed in ESD combined with CSM-PLT group was faster than that of conventional group regardless of lesion location (11.8 mm^2^/min vs. 8.5 mm^2^/min for body, *P* = 0.002, 10.5 mm^2^/min vs. 7.1 mm^2^/min for fundus, *P* = 0.011). The resection speed was 31.6% faster in ESD combined with CSM-PLT group than in conventional group for lesion located in anterior wall (10.0 mm^2^/min vs. 7.6 mm^2^/min, *P* = 0.014) and 54.3% faster for lesion located in lesser curvature (12.5 mm^2^/min vs. 8.1 mm^2^/min, *P* = 0.004). The difference of resection speed for lesion located in posterior wall and greater curvature was not significant (*P* = 0.096 and *P* = 0.330, respectively). For LGIN and HGIN, the resection speed of ESD combined with CSM-PLT was significantly faster than conventional ESD (*P* = 0.001 and *P* = 0.042).

**Table 3:** Subgroup analysis

Resection speed (mm^2^/min)	ESD combined with CSM-PLT (n = 42)	Conventional ESD (n = 65)	P value
Body	11.8(10.0–15.0)	8.5(5.9–11.4)	**0.002** ^**c**^
Fundus	10.5(8.6–14.6)	7.1(4.9–10.5)	**0.011** ^**c**^
Position			
Anterior wall	10.0(9.4–13.4)	7.6(4.2–10.0)	**0.014** ^**c**^
Posterior wall	11.3(7.3–15.0)	7.6(5.7–11.9)	0.096^c^
Greater curvature	10.7(9.5–23.6)	9.1(6.1–17.2)	0.330^c^
Lesser curvature	12.5(10.2–16.2)	8.1(6.1–11.8)	**0.004** ^**c**^
Histology			
LGIN	10.4(8.8–14.4)	7.9(5.6–10.5)	**0.001** ^**c**^
HGIN	13.3(10.2–15.0)	9.1(6.8–10.8)	**0.042** ^**c**^
Adenocarcinoma	11.3(9.7–15.0)	14.7(6.7–15.5)	1.000^c^

^c^ Mann-Whitney test

LGIN, low grade intraepithelial neoplasia; HGIN, high grade intraepithelial neoplasia

*Note*The significance of bold emphasis is P ＜ 0.05

## Discussion

In this retrospective study, we compare the feasibility and safety of ESD combine with CSM-PLT and conventional ESD for the treatment of patients with LGIN or HGIN or EGC at gastric fundus and body. Our results showed that ESD combine with CSM-PLT increased resection speed compared with conventional ESD, without decreasing the rate of *en* bloc resection or increasing the complications. Besides, there were no significance differences in hospital stays and operation costs between two groups. One intra-operative perforation was observed in ESD combine with CSM-PLT group, while two intra-operative perforation was developed in conventional ESD group. One post-operative bleeding was observed in ESD combine with CSM-PLT group and conventional group respectively. There were 20 patients developed intra-operative bleeding in ESD combined with CSM-PLT group and 37 in conventional ESD group. Fortunately, endoscopy intervention was successfully performed in these patients. However, the difference of complication was not significant.

Gastric cancer is the second most common malignant tumor in China [20]. Early detection, diagnosis and treatment are key to improve the survival rate for the patients with gastric cancer. With the development of endoscopic technique, ESD has been an important method for the therapy of early gastric cancer [1, 9]. It is a less invasive operation that does not affect the quality of life, and achieve higher *en* bloc resection rate for the lesions with large size or irregular shape [1, 3]. However, ESD is still challenging because of time-consuming and complications such as bleeding and perforation, especially the lesions in the upper third of the stomach [2, 6, 21]. Studies have shown that the lesions in the upper or middle third of the stomach is a risk factor of perforation, because endoscopy is harder to perform, and the gastric wall is thinner than that of the lower third [6, 22, 23]. In recent years, various traction methods have emerged, and have their own advantages or disadvantages, yet none of them has been widely used by endoscopists [9].

Recently, a multicenter, randomized controlled trial was performed to assess the efficacy compared CSM-PLT with conventional ESD for gastric lesions treatment [24]. It has found that the procedure duration was significantly shorter in the CSM-PLT group than in the conventional ESD group, whereas there were no significance differences in terms of *en* bloc, R0 resection rate and complications between CSM-PLT group and conventional ESD group [24]. In this study although the procedural duration of ESD combine with CSM-PLT was longer than conventional ESD, the resection speed in ESD combine with CSM-PLT group was significantly faster than the conventional group. The reason for this result may be that the specimen area in ESD combine with CSM-PLT group was larger than the conventional group. Therefore, the same specimen area, CSM-PLT may shorten the procedural duration compared to conventional ESD. Previous study [25] indicated that longer procedure duration was the risk factor of endoscopic perforation. In other words, increasing the operation speed means reduce the risk of complication, as well as alleviates the fatigue of endoscopists. Besides, subgroup analysis showed that CSM-PLT significantly improved the resection speed in both gastric fundus and body, especially for lesions in anterior wall and lesser curvature, while there was no significant difference of resection speed for lesion in greater curvature and posterior wall. In fact, ESD of the greater curvature of the upper and middle part of the body is more difficult to operate. The result may be due to the small sample size. It is well known that gastric antrum is the most common site for gastric cancer, while it is less common in gastric body and fundus, and it may contribute to the small sample size. Therefore, it is necessary to conduct a study with larger samples in the future.

CSM-PLT has several of advantages. First, CSM-PLT was used a hemoclip to grasp the dissected mucosal flap, so that which can not only control the traction tension, but also pull or push the lesion through the snare, thus fully exposing the submucosa and obtaining a good cutting field. Because of the good visualization, it was able to clearly identify the layers of submucosa and the vascular distribution, which contributes to improving the resection speed, achieving a higher *en* bloc resection rate and reducing the incidence of complications. A safety operation promotes the recovery of patients and helps to reduce the length of hospital stays, as well as alleviates the fatigue of endoscopists. Therefore, CSM-PLT may be superior to internal traction. Second, the device of CSM-PLT is simple and easy to obtain. We used a hemoclip and snare as auxiliary devices during ESD, which were easily accessible. And it only takes about five cases training to master the technique. In contrast, the “yo-yo technique” [16] requires additional forceps to grasp and orientate the snare, and the process is difficult and may damage the gastric mucosa. While pocket creation method [26, 27] requires a small-caliber-tip transparent hood to provide traction, which is not equipped in every hospital. Third, CSM-PLT can be applied to different sites. Yoshida et al. [28] have demonstrated that CSM-PLT can be applied to gastric ESD. In addition, it was also use for the treatment of colon [19], rectum [29, 30] and esophagus lesions [18], and similarly shortened the procedural duration. Hence, CSM-PLT is a simple, convenient and feasible traction method.

This study had several limitations. First, this was a retrospective, single-center study, and there may be selection bias. Second, the sample size was small, which may contribute to the statistical difference of specimen area. Therefore, a prospective, muti-center study with a large number of patients is needed in the future. Third, we cannot blind the operators to the operation method, making it impossible to ignore performance bias. Luckily, our two endoscopists are very experienced with mature operation technique. Fourth, we only compared ESD combine with CSM-PLT with conventional ESD, not with other traction methods. CSM-PLT is still in the development stage, and we cannot draw a conclusion that it is superior to other traction methods for the time being.

## Conclusions

CSM-PLT is a feasible and safe traction method for the treatment of LGIN or HGIN or EGC at gastric fundus or body. Compared with conventional ESD, CSM-PLT can shorten procedural duration.

## Data Availability

All data and materials included in this study are available upon request by contact with the corresponding author.

## Abbreviations

LGIN: Low grade intraepithelial neoplasia
HGIN: High grade intraepithelial neoplasia
ESD: Endoscopic submucosal dissection
EGC: Early gastric cancer
CSM-PLT: Clip-and-snare method with a pre-looping technique
SD: standard deviation
IQR: interquartile range
